# Monitoring the CO_2_ Emission Trajectory and Reduction Effects by ETS and Its Market Performances for Pre- and Post-pandemic China

**DOI:** 10.3389/fpubh.2022.848211

**Published:** 2022-02-17

**Authors:** Kun Luo, Aidi Xu, Rendao Ye, Wenqian Li

**Affiliations:** ^1^Department of Digital Economy and Management, Alibaba Business School, Hangzhou Normal University, Hangzhou, China; ^2^Department of Logistics Management, School of International Business, Zhejiang Yuexiu University, Shaoxing, China; ^3^Statistics Department, School of Economics, Hangzhou Dianzi University, Hangzhou, China

**Keywords:** COVID-19 pandemic, CO_2_ emission reduction, emission trading scheme (ETS), pilot market performances, propensity-score-matching difference-in-differences (PSM-DID)

## Abstract

The COVID-19 pandemic has caused great shocks on economic activities and carbon emissions. This paper aims to monitor the CO_2_ emission trajectory in China before and after the pandemic outbreak, and analyze the emission reduction effects by ETS and its market performances, which are important determinants underlying the trajectory and key drivers for emission reductions. We firstly find out a rather consistent trajectory of CO_2_ emissions in pre- and post-pandemic China over a 2-year time horizon, using the near-real-time datasets of daily CO_2_ emissions by Carbon Monitor and applying the Cox-Stuart trend test and mean equality test. We then examine the emission reduction effects by China's carbon ETS and its pilot market performances, using the methodologies of DID and PSM-DID as well as pre-pandemic region-level emission datasets by CEADs. Furthermore, it's found that the ETS pilot markets, which are immature with defects, have been performing more vulnerably in terms of liquidity and transaction continuity under pandemic shocks, thus undermining the emission reduction effects by ETS. These findings are providing insights into further mechanism design of the carbon ETS to the end of steady emission reductions even under shocks for post-pandemic China. It's of particular importance now that the nationwide market has been launched and needs to be enhanced based on lessons learned.

## Introduction

The COVID-19 pandemic has caused tremendous losses of human lives and wellbeing ever since its first outburst at the beginning of 2020. Enforced lockdowns and voluntary cut-down in inessential activities led to huge reductions in energy consumption and recession of the global economy. Anthropic greenhouse gas emissions dropped drastically all over the world (1–3). In China where the virus was first spotted and contained with strong measures, CO_2_ emissions fell sharply by more than −10% in 2020Q1 over 2019Q1 (4–6). In this first wave of the pandemic, daily global CO_2_ emissions decreased by −17% on average by early April 2020 compared with the mean in 2019, equivalent to levels last seen in 2006. At the peak, daily emission reductions in individual countries averaged to −26%. Median of the estimates from a number of studies show that global emissions fell by −7% in 2020 compared to 2019, including reductions by −12% in the US, −11% in the EU, −9% in India, and −1.7% in China. The year 2020 witnessed the largest absolute annual decline in CO_2_ emissions ever recorded, as well as the largest relative fall since World War II (7–10).

This decline in emissions seemed to be temporary though, as mostly due to reduced economic activities and energy use during lockdowns. Le Quéré (10) devised a confinement index on a scale of 0 to 3 to quantify the levels of restrictions to normal activities during the pandemic, and estimated its effects on CO_2_ emissions for six sectors of the economy. Their analysis, covering 85% of the world population and 97% of global CO_2_ emissions, found that confinement had been significantly determining the reduction in emissions for January through April 2020. This strong correlation between containment and emission reductions would herald a resurgence of emissions all over the world when containment gets relaxed (11). Emissions are expected to return to their normal trajectory and even make things worse, unless more profound changes are induced toward a cleaner energy structure and less carbon-intensive economic system (12).

Take China for instance. As the crisis unfolded since December 2019, the Chinese government has implemented forceful containment measures, including shutting down factories, schools and cities. The economy was shocked heavily in late January 2020, and CO_2_ emissions fell sharply by −25% over a 4-week period commencing 3 February 2020 after the lunar new-year break, compared with usual levels (13). Estimates of China's daily CO_2_ emissions further revealed that the monthly variations between 2020 and 2019 were −18.4 and −9.2% in February and March, respectively (14). Then as China slowly emerged from the first wave, work and production resumed, energy use resurged, and emissions began to return to normal. By late March when lockdowns gradually lessened, the pandemic effects on emissions had begun to diminish. Monthly variations between 2020 and 2019 turned out to be +0.6% in April and +5.4% in May, indicating that CO_2_ emissions have returned to and even bounced higher than their pre-pandemic levels. Similar findings have been reported by several studies (15, 16).

As after previous financial crises, there are chances that governments' stimulus in response to the disruption generate a retaliatory rebound in emissions. It was predicted that global CO_2_ emissions could exceed the pre-crisis levels over a 2-year horizon commencing 2020Q1, even if another wave of pandemic was to occur within a year. The bouncing trend has been observed even in countries renewing containment measures due to pandemic rebounds (17–21). In this very likely event of emission resurgence worldwide, prospects of meeting the objectives of global warming control to 1.5–2°C above pre-industrial levels under the Paris Agreement could seem worsened. Thus, apart from observing the short-run decline and rebound, it is high time to probe further into the pandemic impacts on carbon emissions and their drivers underlying. Whether the pandemic is changing the emission trajectory and the main driving forces for emission reductions are key questions in concern.

This paper aims to monitor the CO_2_ emission trajectory in China before and after the pandemic, and analyze the emission reduction effects by ETS and its market performances, which are important determinants underlying the emission trajectory and key drivers for emission reductions. We firstly examine the pre- and post-pandemic trajectory of CO_2_ emissions in China over a 2-year time horizon, using the daily CO_2_ emission datasets by Carbon Monitor and applying the Cox-Stuart trend test and mean equality test. We then look into the emission reduction effects by China's carbon ETS, using the methodology of PSM-DID and pre-pandemic region-level emission datasets by CEADs. Considering the ETS is market-based and pilot markets have been experiencing shocks from the pandemic, we further study the emission reduction effects by pilot markets, which are immature with ever-existing defects and have been performing more vulnerably in terms of liquidity and transaction continuity under pandemic shocks than normal times. These findings are providing insights into the emission reductions by ETS and its market performances with further mechanism design for post-pandemic China.

## Pre- and Post-pandemic Emission Trajectory in China

### Data and Descriptive Statistics

**Figure 1:**
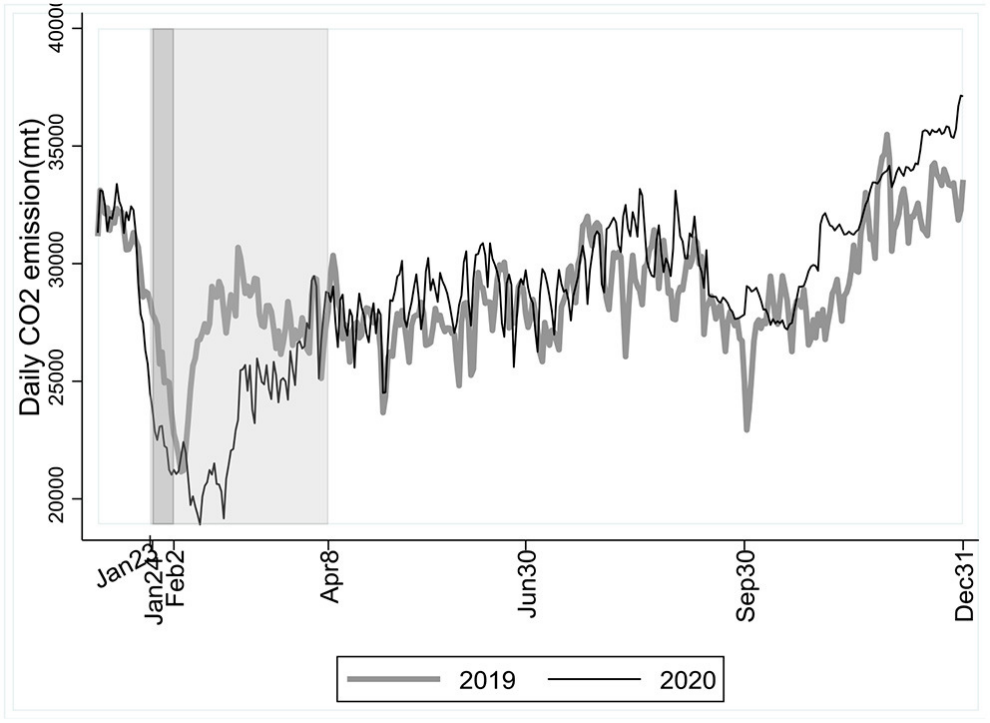
China's daily CO_2_ emissions in 2019 and 2020, based on datasets by Carbon Monitor covering emissions from sectors of power, industry and cement production, ground transport, aviation, international shipping, residential, and commercial buildings. The dark-shaded area illustrates the lunar new-year break, while the light-shaded area illustrates the lockdown of Wuhan City, quite a representative of the first wave of the pandemic in China.

We use the near-real-time sector-specific region-level estimates of daily CO_2_ emissions based on activity data by Carbon Monitor. The dataset covers emissions from fossil fuel use and industry including process emissions from cement production. Figure 1 illustrates the daily CO_2_ emissions in China over 2 years commencing 1 January 2019. Despite of severe shocks in the short run, the trajectory of emissions was consistent before, during and after the pandemic outbreak over the 2-year time horizon.

The COVID-19 was first sporadically spotted in late December of 2019 and soon evolved into an unprecedented nationwide pandemic by late January 2020, represented by the lockdown of Wuhan City on 23 January 2020 which lasted for 76 days. The Chinese government immediately imposed forceful containment measures, including calling off economic and social activities especially during the lunar new-year break from 24 January till 2 February 2020. Normally CO_2_ emissions stay low with the economy during the lunar new-year break, the year 2020 was without exception, only to see a drop more acute and lasting than pre-pandemic times. Average daily emissions declined by −3.98% during the lunar new-year break in 2020 compared with the break in 2019. The economy was shocked severely in 2020Q1, and CO_2_ emissions didn't recover after the break as usual. Compared to pre-pandemic 2019 when daily emissions bounced back to pre-break levels only 4 days after the beginning of the break, daily emissions during the break in 2020 returned to pre-break levels until 38 days later. The changes in CO_2_ emissions before and during the first wave of the pandemic were apparent.

The emission trajectory turned out to be quite stable though. Fluctuations in daily emissions before and after the pandemic outbreak were roughly synchronous over a longer time horizon. With the rapid economic recovery, the short-run impacts on emissions had diminished by the end of March, and daily emissions have returned to their normal trajectory ever since. Table 1 gives the descriptive statistics of China's daily CO_2_ emissions during same periods between 2019 and 2020. The mean and median (P50) of daily emissions are not much different in specific periods of 2020 compared to 2019. The standard deviation (SD) and coefficient of variation (CV) from January to August 2020 compared to the same period in 2019 indicate changes related to the pandemic. However, such changes began to diminish since September, and have almost vanished throughout the rest of 2020, with the SD and CV being quite close to the levels in 2019.

**Table 1:** Descriptive statistics of China's daily CO_2_ emissions in 2019 and 2020.

**Period**	**Mean**	**P50**	***SD***	***CV***
20190101-20190131	29802.335	30701.864	2483.063	0.083
20200101-20200131	28694.036	31301.914	4210.001	0.147
20190201-20190831	27882.246	27841.723	1870.095	0.067
20200201-20200831	27443.480	28378.704	3225.738	0.118
20190901-20190930	28358.785	28196.294	1406.607	0.050
20200901-20200930	29446.249	28630.508	1537.701	0.052
20191001-20191231	30172.647	29718.501	2719.407	0.090
20201001-20201231	31927.276	31814.731	2885.190	0.090

### Cox-Stuart Trend Test

We apply two diagnostic tests to verify the consistency in the trajectory of China's daily CO_2_ emissions before and after the pandemic. We firstly use the Cox-Stuart trend test (22, 23) for the following two sets of hypotheses.

(*HypothesisTesting1*)

H_0_: no downward trend in data

H_1_: downward trend in data

(*HypothesisTesting2*)

H_0_: no upward trend in data

H_1_: upward trend in data

Let *X* denote a data set with size *n*, namely *X* = {*x*_1_, *x*_2_, …, *x*_*n*_}. Take *x*_*i*_ and *x*_*i*+*c*_ to form a pair of data (*x*_*i*_, *x*_*i*+*c*_), *i* = 1, 2, …. When *n* is an even number, *c* groups of data pair are generated; otherwise, *c-1* groups of data pair are generated, where


$$c=\{\begin{array}{cc} \frac{n}{2} & \text{if n is an even number}\\ \frac{\left(n+1\right)}{2} & \text{otherwise} \end{array}$$

Let *D*_*i*_ = *x*_*i*_ − *x*_*i*+*c*_. *S*^+^ and *S*^−^ are the numbers of positive or negative number of *D*_*i*_, respectively. *K* is the test statistic, and α is the nominal significance level. Further, when verifying *HypothesisTesting1*, then *K* = *S*^−^; when verifying *HypothesisTesting2*, then *K* = *S*^+^.It is easy to prove that if the *H*_0_ in the above hypothesis testing problems is true, then *K* follows the binomial distribution *b*(*m*, 0.5), where *m* = *S*^+^+*S*^−^. Finally, when considering *HypothesisTesting1*, then *p* = Pr(*S*^−^ ≤ *s*^−^); when considering *HypothesisTesting2*, then *p* = Pr(*S*^+^ ≤ *s*^+^), where *s*^+^and *s*^−^, respectively, represents the observed values of *S*^+^ and *S*^−^. If *p* < α, then we reject *H*_0_ at the nominal significance level α.

Table 2 gives the results of Cox-Stuart trend test for four specific periods between 2019 and 2020. For January and September, there seemed to be a downward trend in daily CO_2_ emissions as illustrated in Figure 1, and we hereby consider *HypothesisTesting1*. Results show that the *p*-values of the Cox-Stuart trend tests in January 2019 and 2020 are equal (namely, 3.05e-05), and *H*_0_ is rejected at the nominal significance level of 5%. Daily emissions in January 2020 and 2019 were both showing the downward trend, and so were emissions in September 2019 and 2020.

**Table 2:** Cox-Stuart trend test.

**Period**	**Trend**	***S^**−**^***	***S^**+**^***	***K*-value**	***p*-value**
20190101-20190131	Downward	0	15	0	3.05e-05
20200101-20200131	Downward	0	15	0	3.05e-05
20190201-20190831	Upward	78	28	28	6.26e-07
20200201-20200831	Upward	101	5	5	1.31e-24
20190901-20190930	Downward	1	14	1	4.88e-04
20200901-20200930	Downward	0	15	0	3.05e-05
20191001-20191231	Upward	46	0	0	1.42e-14
20201001-20201231	Upward	46	0	0	1.42e-14

For February through August, and for October through December, daily CO_2_ emissions seemed to be on the rise on the whole, and we consider *HypothesisTesting2*. The *p*-values for February through August in 2019 and 2020 are 6.26e-07 and 1.31e-24, respectively, and *H*_0_ is rejected at the nominal significance level of 5%. Daily emissions from February to August in 2020 and 2019 were both showing the upward trend. Similar results are observed for the periods from October to December in 2019 and 2020.

### Mean Equality Test

**Figure 2:**
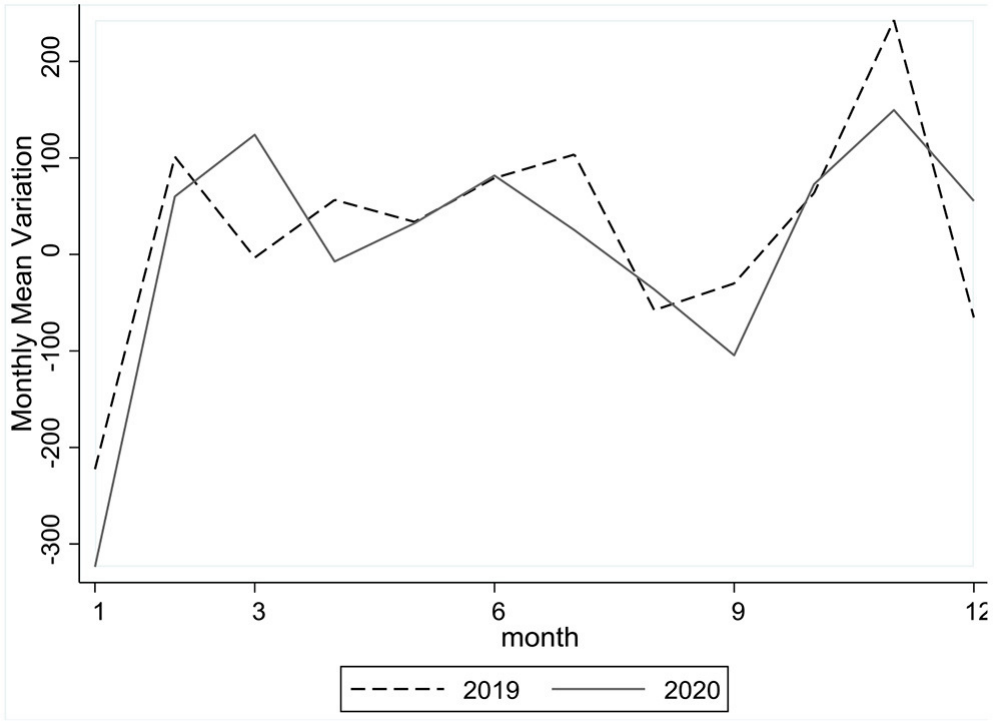
Monthly mean variations in CO_2_ emissions between 2019 and 2020.

Based on the Cox-Stuart trend test indicating downward or upward trends of daily CO_2_ emissions in certain periods between 2019 and 2020, we further apply the mean equality test to verify the consistency between trends with the same direction. Calculate the monthly mean variations in emissions as sums of daily variations divided by the numbers of days within a month, and Figure 2 shows the contours of monthly mean variations between 2019 and 2020. We then conduct the equality test to examine whether the two contours are overlaps. With the *p*-value being 0.81, the null hypothesis (*H*_0_: two contours are overlaps) was not rejected. This mean equality test combined with the Cox-Stuart test verifies that daily emissions in the same periods between 2019 and 2020 shared the same downward or upward trends with variations statistically indifferent from each other.

For the time being, we find no signs of significant changes in the trajectory of China's daily CO_2_ emissions, even though there were abrupt shocks in the first wave of the pandemic. This finding is based on near-real-time estimates of pre- and post-pandemic emissions from fossil fuel use and industry using the same methodology by Liu et al. (4, 14). In other words, when measured on the same scale, CO_2_ emissions in China before and after the pandemic were following a consistent trajectory over a 2-year time horizon. Even so, it's necessary to look further into the pandemic impacts on forces underlying the emission trajectory and driving for emission reductions, before we can get a clearer picture about the pandemic impacts on CO_2_ emissions.

## Pre-pandemic Emission Reduction Effects by ETS

Amongst the driving forces of carbon emissions and their reductions (24–27), the emission trading scheme (ETS) has been regarded as of particular importance. As a market-based environmental regulation mechanism, carbon ETS works on firms' incentives and behavior in emission abatement, including cleaner technology innovation and application, through prices of emission permits or allowances tradable on the market. Being a key driver for emission reductions, ETS's reduction effects have been empirically analyzed with a variety of methodologies (28–31). In this section we apply the methodologies of propensity-score-matching (PSM) and difference-in-differences (DID) to examine the reduction effects of China's carbon ETS based on pre-pandemic data. The results will hopefully provide us with insights into the emission reductions effects by this key driver for post-pandemic China, which are discussed in the next section.

### PSM-DID Methodology

#### Baseline Model

China's carbon ETS pilots have been running several compliance cycles since their successive launch from 2013 to 2016 in eight municipalities and provinces of Shanghai, Beijing, Tianjin, Chongqing, Guangdong (with Shenzhen included herein), Hubei, and Fujian. Although a nationwide ETS covering the power generation industry was introduced in 2017 and began its the first compliance cycle starting from 2021, the region-specific pilots with a broader coverage of sectors and enterprises are still going to play important roles in China's ETS. We take these pilot regions as treatment and the other provinces in mainland China (except for Tibet due to missing data) as control, and use the DID model as in Equation (1) to study the effects on CO_2_ emissions by ETS policies.


(1) $$\begin{array}{l} \ln\;\left(emission_{it}\right)=\beta_{0}+\beta_{1}DID_{it}+province_{i}+year_{t}\\ +\Sigma \lambda control_{it}+\varepsilon_{it} \end{array}$$

where ln (*emission*_*it*_) denotes the log of CO_2_ emissions for region *i* in year *t*. *DID*_*it*_ is the interaction term of *pilot*_*i*_ × *post*_*it*_. *pilot*_*i*_ is the group dummy, *pilot*_*i*_ = 1 if *i* ∈ *treatment* and *pilot*_*i*_ = 0 if otherwise. *post*_*it*_ is the time dummy, *post*_*it*_ = 1, if *t* = 2013 for Beijing, Tianjin, Shanghai, and Guangdong, if *t* = 2014 for Chongqing and Hubei, if *t* = 2016 for Fujian; and *post*_*it*_ = 0 if otherwise. Then, the coefficient β_1_ of *pilot*_*i*_ × *post*_*it*_ indicates the average change in the log of CO_2_ emissions in pilot regions relative to that in non-pilot regions post policy implementation. *province*_*i*_ denotes the region-fixed effects on CO_2_ emissions from unobserved region-specific time-invariant determinants, while *year*_*t*_ denotes the time-fixed effects.

A set of control variables denoted by *control*_*it*_ captures the main determinants of CO_2_ emissions.

*ln(realGDPpc)* and *ln(popden)*, respectively, denote the log of real GDP per capita calculated at prices in 2005 and population density, controlling impacts on emissions related to the economic scale and population agglomeration.

*indshare* and *coalshare* denote the proportions of added value from the secondary industry to GDP, and coal consumption to total energy consumption, respectively, representing the structure of the economic and energy system.

Energy consumption intensity denoted by *ecintensity*, calculated as consumption of standard coal equivalent per unit of real GDP, could be regarded as a proxy for technology advancement related to energy conservation and emission reduction.

*urbanshare, eximshare, rdshare*, and *indpoinvestshare* denote the proportions of urban population to total population, trade value to GDP, industrial enterprises' expenditures on R&D to GDP, and investment completed in the treatment of industrial pollution to GDP, respectively, controlling impacts on emissions related to urbanization, trade, technology innovation and environmental regulation.

#### Descriptive Statistics

We use the region-level CO_2_ emission inventories in China by CEADs, and datasets on regional economy, energy use, and environment by NBS. See Section Data Availability Statement for details. Table 3 summarizes the descriptive statistics of variables across treatment and control groups from 2005 to 2019. On average, the absolute magnitude of CO_2_ emissions in pilot regions is lower than that in non-pilot regions by almost 20%. It's reasonable with the mean of *indshare, coalshare*, and *ecintensity* being smaller and the mean of *rdshare* being greater in pilot regions, indicating positive effects in reducing CO_2_ emissions in an economic structure featured by less-intensive fossil fuel use and greater investment on technology innovation, which more than offset the emissions from a larger scale of economic output, population agglomeration and urbanization.

**Table 3:** Descriptive statistics of variables by group.

**Variables**	**Pilot (*****N*** **=** **105)**				**Non-pilot (*****N*** **=** **345)**			
	**Mean**	**P50**	**SD**	**CV**	**Mean**	**P50**	**SD**	**CV**
*ln(emission)*	5.259	5.183	0.537	0.102	5.482	5.552	0.824	0.150
*ln(realGDPpc)*	1.479	1.557	0.531	0.359	0.803	0.847	0.512	0.637
*ln(popden)*	6.577	6.421	0.850	0.129	5.091	5.344	1.176	0.231
*indshare*	41.173	44.934	10.173	0.247	43.695	43.865	7.445	0.170
*coalshare*	60.578	61.722	19.104	0.315	105.473	99.759	39.612	0.376
*ecintensity*	0.772	0.732	0.326	0.421	1.511	1.284	0.816	0.540
*urbanshare*	69.471	64.243	15.195	0.219	49.432	49.734	9.470	0.192
*eximshare*	73.687	71.803	49.951	0.678	18.611	12.504	16.991	0.913
*rdshare*	1.337	1.262	0.591	0.442	0.808	0.687	0.498	0.616
*indpoinvestshare*	0.108	0.076	0.103	0.955	0.176	0.143	0.151	0.858

#### Propensity Score Matching

The *DID* estimates indicate the emission reduction effects induced by ETS, with the control group providing effective counterfactual changes to emissions of the pilot regions. It's important to identify parallel trends in emissions across treatment and control groups over time, before we attribute variability across groups to the effects induced by ETS policies rather than pre-existing time trends. Since the ETS pilots were not randomly selected, it might not be appropriate if we take all non-pilot regions as control. For this concern, we use the Probit model as in Equation (2) to calculate propensity scores and match pilot regions to non-pilot regions that have similar characteristics in covariates of CO_2_ emissions. Table 4 gives the results.


(2) $$\begin{array}{l} P\left(control_{i}\right)=\mathrm{Pr}\left(pilot_{i}=1|control_{i}\right)=E\left(pilot_{i}|control_{i}\right) \end{array}$$

**Table 4:** Probit regression.

**Pilot**	**Coefficient**	***t*-Value**
*ln(realGDPpc)*	−3.432***	−3.55
*ln(popden)*	2.529***	3.94
*indshare*	0.211***	5.58
*coalshare*	−0.087***	−5.64
*ecintensity*	−1.791***	−2.70
*urbanshare*	0.240***	5.29
*eximshare*	−0.039***	−4.24
*rdshare*	−1.230**	−2.35
*indpoinvestshare*	2.903*	1.82
Constant	−23.737***	−5.07
Observations	450	
Pseudo *R*^2^	0.675	

***p < 0.01, **p < 0.05, *p < 0.1.

Then, we match pilot and non-pilot regions year by year, using the estimated propensity scores and a most-commonly-used matching algorithm, the *k*-nearest neighbor matching (NNM) within radius, with *k*=2 and $radius=0.5{\hat{\sigma }}_{ps}$, where ${\hat{\sigma }}_{ps}$ denotes the SD of estimated propensity scores. Table 5 reports the differences in the mean values of variables across treatment and control groups after matching. For all variables used in this study, mean values across groups are much more balanced after matching. Results of *t*-test further show that for most variables the mean values across groups are insignificantly different, suggesting the validity of matching.

**Table 5:** Mean of variables before and after PSM (2-NNM within radius of 0.174).

**Variables**	**Sample**	**Treatment**	**Control**	**Difference**	***p*-Value of *t*-Test**
*ln(emission)*	Unmatched	5.259	5.482	−0.223	0.001
	Matched	5.350	5.442	−0.092	0.577
*ln(realGDPpc)*	Unmatched	1.479	0.803	0.676	0.000
	Matched	1.031	0.946	0.085	0.395
*ln(popden)*	Unmatched	6.577	5.091	1.486	0.000
	Matched	6.050	5.903	0.146	0.185
*indshare*	Unmatched	41.173	43.695	−2.523	0.020
	Matched	46.083	47.751	−1.668	0.268
*coalshare*	Unmatched	60.578	105.473	−44.895	0.000
	Matched	74.456	79.367	−4.910	0.168
*ecintensity*	Unmatched	0.772	1.511	−0.739	0.000
	Matched	0.969	0.960	0.009	0.897
*urbanshare*	Unmatched	69.471	49.432	20.038	0.000
	Matched	58.279	52.680	5.600	0.010
*eximshare*	Unmatched	73.687	18.611	55.076	0.000
	Matched	57.077	38.544	18.534	0.059
*rdshare*	Unmatched	1.337	0.808	0.529	0.000
	Matched	1.014	1.039	−0.026	0.801
*indpoinvestshare*	Unmatched	0.108	0.176	−0.068	0.000
	Matched	0.138	0.111	0.027	0.235

*Matched sample size: N = 88, with 43 and 45 observations in treatment and control, respectively*.

### PSM-DID Results of Average Treatment Effects

Columns (3)–(8) in Table 6 give the PSM-DID results using NNM within radius of $0.5{\hat{\sigma }}_{ps}$. For reference, we also report the DID baseline regression results as in Columns (1)–(2). The coefficients of *pilot* × *post* under various regression scenarios are all statistically significant and negative, indicating significant effects in reducing emissions by ETS in pilot regions. Moreover, the PSM-DID coefficients (ranging from −0.04 to −0.09) are smaller in magnitude than DID coefficients (−0.13, approximately), indicating that the emission reduction effects by ETS could have been exaggerated if we simply applied the DID methodology without caution. With more reliable estimates after PSM, we calculate the average treatment effects on the absolute magnitude of CO_2_ emissions in pilot regions post ETS implementation, relative to control groups of non-pilot regions (pre- or post-ETS) and pilot regions pre-ETS. Measured with different sizes of matched samples using the same matching algorithm, CO_2_ emissions in pilot regions post ETS are reduced by 4–8.6%.

**Table 6:** PSM-DID results.

**Variables**	***ln(emission)***							
	**DID**		**PSM-DID (*****k*****-NNM within radius of** $0.5{\hat{\sigma }}_{ps}$**)**					
			***k*** **=** **2**		***k*** **=** **4**		***k*** **=** **6**	
	**(1)**	**(2)**	**(3)**	**(4)**	**(5)**	**(6)**	**(7)**	**(8)**
*Pilot × post*	−0.139***	−0.134***	−0.041*	−0.042*	−0.073***	−0.078***	−0.091***	−0.090***
	(−6.87)	(−6.69)	(−1.62)	(−1.74)	(−3.44)	(−3.76)	(−4.64)	(-4.55)
Controls	All*^*a*^*	Yes*^*b*^*	All*^*a*^*	Yes*^*b*^*	All*^*a*^*	Yes*^*b*^*	All*^*a*^*	Yes*^*b*^*
Province-fixed effects	Yes	Yes	Yes	Yes	Yes	Yes	Yes	Yes
Year-fixed effects	Yes	Yes	Yes	Yes	Yes	Yes	Yes	Yes
Constant	−3.380***	−3.637***	4.829**	3.349***	5.960***	3.549***	7.378***	8.686***
	(−4.66)	(−5.10)	(2.27)	(13.64)	(3.15)	(16.11)	(4.15)	(5.36)
Observations	450	450	88	88	111	111	124	124
R^2^	0.129	0.125	0.598	0.451	0.559	0.495	0.375	0.221
Effects on *emissions^*c*^*	−12.985	−12.508	−4.009	−4.078	−6.995	−7.466	−8.727	−8.567

*t-values in parentheses*.

***p < 0.01, **p < 0.05, *p < 0.1.

a,b *All the control variables included and only statistically significant ones included, respectively*.

c *Effects on the absolute magnitude of CO_2_ emissions*.

### Robustness Checks

#### Parallel Trend Test

**Figure 3:**
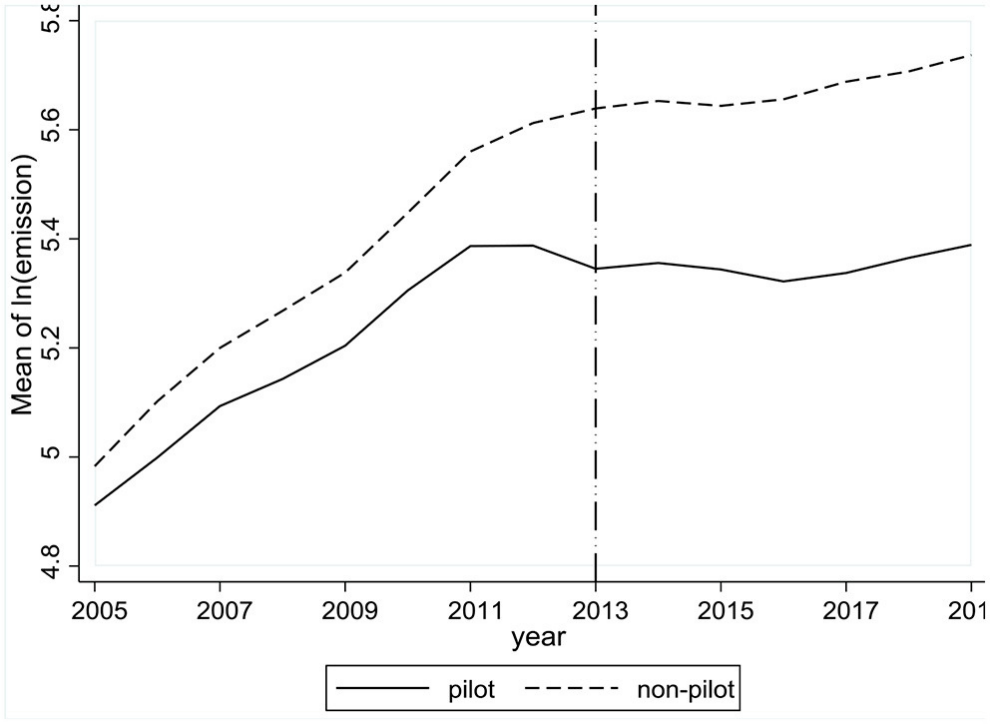
Year-to-year average of *ln(emission)* within groups before and after ETS.

We apply two diagnostic tests for parallel trend across treatment and control groups. Firstly, we calculate the average *ln(emission)* within each group year by year from 2005 to 2019. Figure 3 shows similar upward trends in emissions over time across pilot and non-pilot regions before 2013, indicating that those regions would have similar trends after 2013 if there weren't any ETS policies.

To further verify parallel trend, we follow the methods by Chen et al. (32) and estimate the model as in Equation (3).


(3) $$\begin{array}{l} \ln\;\left(emission_{it}\right)=\beta_{0}+\beta_{1}DID_{it}+\overset{11}{\underset{k=1}{\sum }}\beta_{k}\left(pilot_{i}\times yearbefore_{it}^{-k}\right)\\ +province_{i}+year_{t}+\sum \lambda control_{it}+\varepsilon_{it} \end{array}$$

where $yearbefore_{it}^{-k}$ is a dummy denoting *k*-year(s) before ETS implementation with *k* = 1, 2, …, 11 covering the years from 2005 to 2015. Then, if the coefficient β_*k*_ of ${\text{pilot}}_{i}\times {\text{yearbefore}}_{it}^{-k}$ is statistically insignificant, we consider pilot and non-pilot regions as having parallel trend before policy implementation.

Table 7 gives the results estimated from the sample after PSM using the algorithm of 2-NNM within radius of $0.5{\hat{\sigma }}_{ps}$. The estimated coefficients of $pilot_{i}\times yearbefore_{it}^{-k}$ (*k* = 1, 2, …, 11) are all statistically insignificant, or statistically not different from 0. This finding verifies that the assumption of parallel trend is not violated, and estimates using the PSM-DID methodology capture the emission reduction effects by ETS.

**Table 7:** Parallel trend test.

**Variables**	***ln(emission)***	
	**(1)**	**(2)**
*Pilot × post*	−0.108*	−0.123**
	(−1.74)	(−2.14)
*Pilot × yearbefore^−1^*	−0.083	−0.104
	(−1.30)	(−1.77)
*Pilot × yearbefore^−2^*	−0.065	−0.064
	(−1.22)	(−1.27)
*Pilot × yearbefore^−3^*	−0.047	−0.043
	(−0.90)	(−0.86)
*Pilot × yearbefore^−4^*	−0.045	−0.047
	(−1.00)	(−1.07)
*Pilot × yearbefore^−5^*	−0.040	−0.037
	(−0.94)	(−0.88)
*Pilot × yearbefore^−6^*	−0.036	−0.033
	(−1.42)	(−1.34)
*Pilot × yearbefore^−7^*	−0.052	−0.044
	(−1.45)	(−1.32)
*Pilot × yearbefore^−8^*	−0.081	−0.076
	(−1.95)	(−1.93)
*Pilot × yearbefore^−9^*	−0.076	−0.072
	(−1.80)	(−1.81)
*Pilot × yearbefore^−10^*	−0.073	−0.057
	(−1.35)	(−1.09)
*Pilot × yearbefore^−11^*	0.007	0.012
	(0.11)	(0.20)
Controls	All*^*a*^*	Yes*^*b*^*
Province–fixed effects	Yes	Yes
Year-fixed effects	Yes	Yes
Constant	4.784*	2.988***
	(1.88)	(16.60)
Observations	88	88
*R* ^2^	0.582	0.490

*t-values in parentheses*.

***p < 0.01, **p < 0.05, *p < 0.1.

*^a,b^Refer to Table 6*.

#### Placebo Test

**Figure 4:**
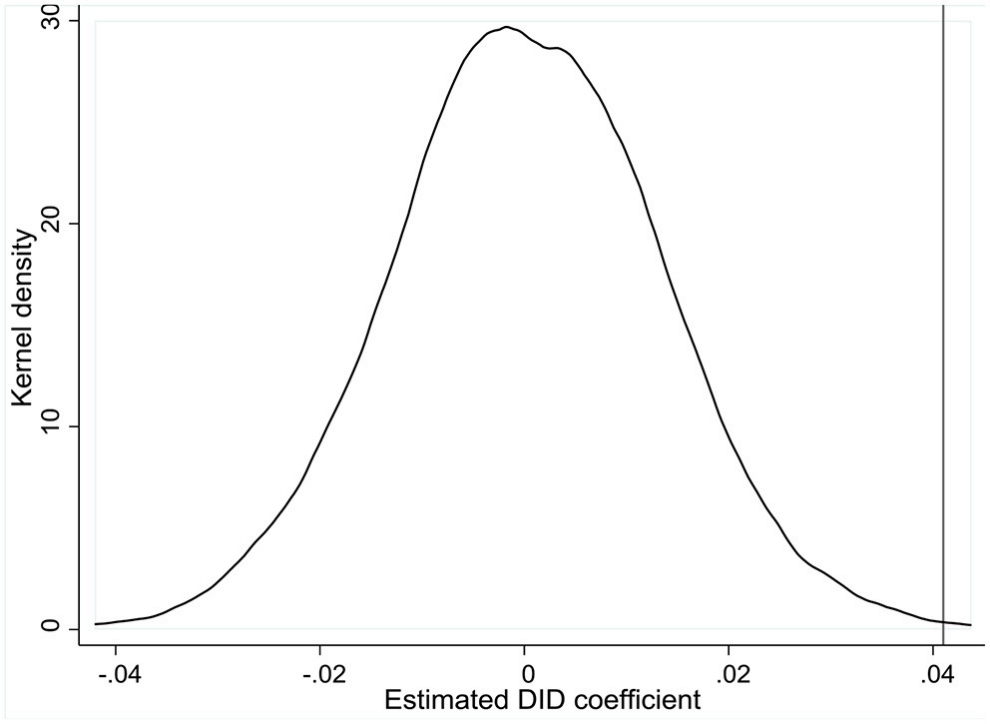
Kernel density of estimated DID coefficients in 1,000 random permutations based on Monte Carlo simulation.

We conduct a placebo test to further check robustness of our estimation results. Based on Monte Carlo simulation, regions and years are randomly selected to generate a virtual treatment group for 1,000 times. With these random permutations, we then use the DID regression to estimate β_1_ as in Equation (1). Figure 4 shows the kernel density of coefficients estimated from counterfactual treatment groups. The estimates from the real treatment group (as reported in Table 6) fall at the tail of the distribution, indicating significant differences in estimates from real and virtual treatment groups. Hence, we exclude the placebo effect and consider the estimates of emission reduction effects by ETS as valid.

## Further Checks on Pilot Market Performances and Post-pandemic Implicatons

### Reduction Effects by Pilot Market Performances

Since the ETS is a market-based mechanism for emission reduction, the reduction effects are bound to vary with the performances of pilot markets [See (33) for a recent review]. To capture the pandemic shocks on emission trading markets and their implications for post-pandemic emission reductions, we hereby estimate as in Equation (4) the emission reduction effects by pilot market performances regarding the trading price, liquidity and transaction continuity.


(4) $$\begin{array}{l} \ln\;\left(emission_{it}\right)=\beta_{0}+\beta_{1}\left(DID_{it}\times performance_{it}\right)\\ +province_{i}+year_{t}+\sum \lambda control_{it}+\varepsilon_{it} \end{array}$$

where *performance*_*it*_ is a set of variables capturing the characteristics of transactions on pilot markets if *i* ∈ *treatment*, and takes the value of 0 if otherwise indicating no market performances whatsoever in non-pilot regions. Following Liu et al. (33) and Cui et al. (34), the set includes the log of yearly average trading prices and volumes of emission allowances, denoted as ln (*price*_*it*_) and ln (*volume*_*it*_), respectively. Considering that a market with greater trading volumes tends to be more active and maintains prospects for assets to be more easily exchanged, we use ln (*volume*_*it*_) to capture the liquidity of pilot markets (35, 36). To represent transaction continuity, we use the counts of continuous trading days and their proportions to total trading days within a year, denoted as *continuitycounts*_*it*_ and *continuityprop*_*it*_, respectively.

Table 8 reports the effects on CO_2_ emissions by pilot market performances, estimated from the datasets as used in the previous section and datasets of emission allowance transactions on various pilot exchanges. In accordance with literature, we find statistically negative coefficient of *DID* × ln(*price*) as in Column (1), indicating the pilot markets are working in emission reductions through the fundamental signals of carbon prices, which reflect marginal abatement costs and provide incentives for emission reductions. Still, to increase the odds of trading prices efficiently reflecting marginal abatement costs, we rely on a moderate liquidity with reasonable trading volumes on pilot markets. As Column (2) reports, the coefficient of *DID* × ln (*volume*) is also statistically negative, indicating the emission trade in pilot regions is working in emission reductions through liquidity of carbon assets. Furthermore, we find statistically negative coefficients of *DID* × *continuitycounts* and *DID* × *continuityprop*. As the proportion of continuous trading days to total trading days within a year increases by 1 unit on pilot markets post implementation of ETS, the log of CO_2_ emissions are estimated to reduce by 0.167%. Apart from the trading prices and liquidity, the continuity of transactions on pilot markets is another important determinant for emission reductions. These findings are of particular importance for us to better understand the post-pandemic emission reductions by ETS.

**Table 8:** Effects by pilot market performances.

**Variables**	***ln(emission)***			
	**(1)**	**(2)**	**(3)**	**(4)**
*DID × ln(price)*	−0.038***			
	(−5.92)			
*DID × ln(volume)*		−0.022***		
		(−6.13)		
*DID × continuitycounts*			−0.001***	
			(−4.46)	
*DID × continuityprop*				−0.167***
				(−5.49)
Controls	Yes	Yes	Yes	Yes
Province-fixed effects	Yes	Yes	Yes	Yes
Year-fixed effects	Yes	Yes	Yes	Yes
Constant	−3.411***	−2.780***	−2.676***	−3.061***
	(−4.75)	(−3.86)	(−3.74)	(−4.29)
Observations	450	450	450	450
*R* ^2^	0.128	0.137	0.136	0.132

*t-values in parentheses*.

***p < 0.01.

### Implications for Post-pandemic Emission Reductions by ETS

As important determinants for emission reduction effects by ETS, pilot market performances, especially in terms of liquidity and transaction continuity, have been experiencing severe shocks during the pandemic.

**Figure 5:**
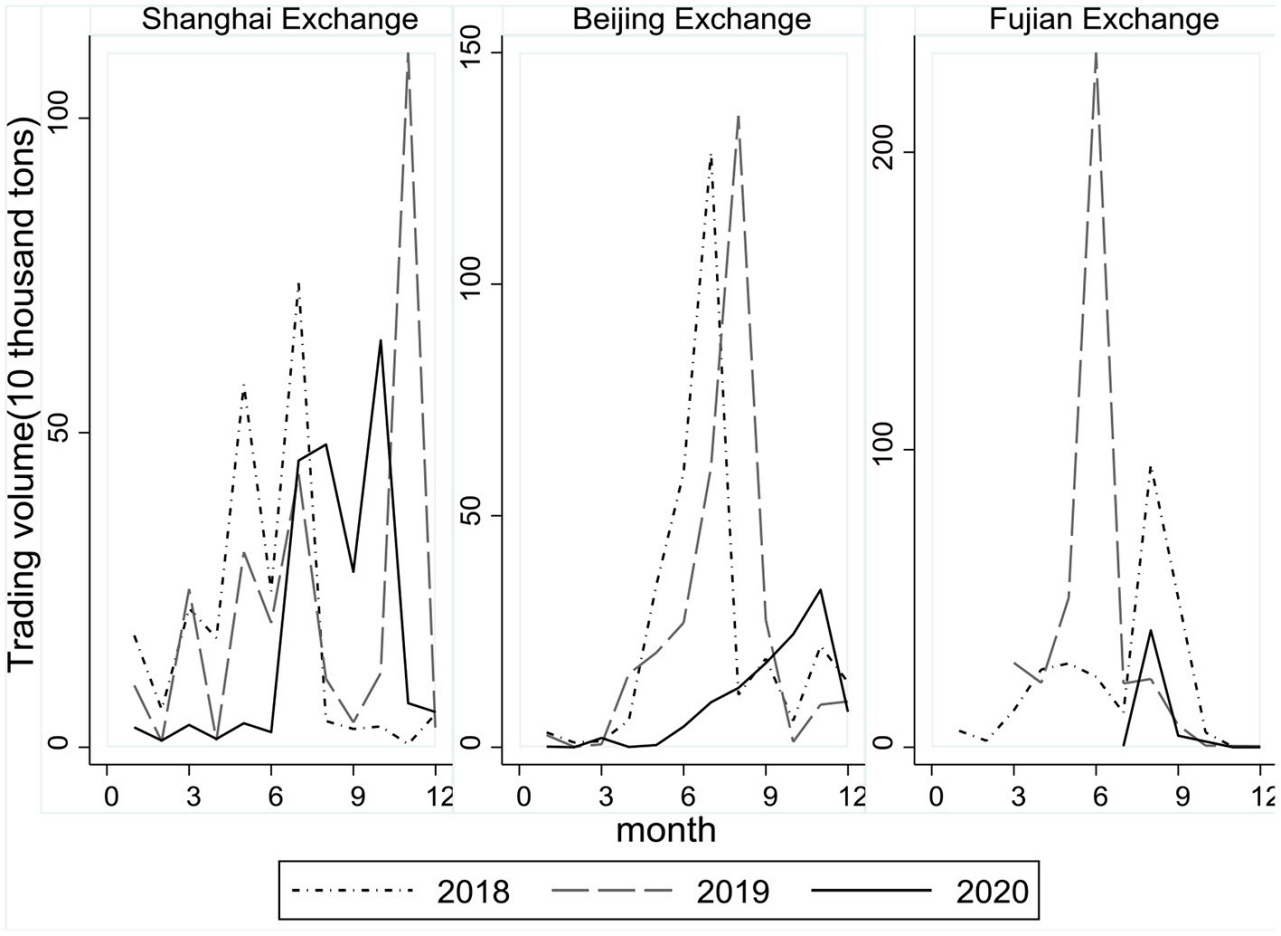
Monthly trading volume and continuity in 2018–2020.

Take the pilots of Shanghai and Beijing for instance. Figure 5 shows that the trading volumes for most months in 2020 were lower than the monthly volumes in pre-pandemic 2018 and 2019 on both pilot markets. A total amount of 2.14 million tons of emission allowances were traded on the Shanghai Exchange in 2020, a fall by −20.78% compared to 2019. During the first wave of the pandemic, the trading volume shrank especially dramatically, falling by −78.3% in 2020Q1 compared to 2019Q1. The Beijing Exchange experienced a very similar decrease in trading volumes, by −63.38% throughout 2020 compared to 2019.

Transactions even halted for months on some markets in 2020. Take the pilot of Fujian for instance. There were no transactions at all for the first 6 months, compared to a total trading volume of 3.34 million tons for the same period in 2019. This transaction halt lasted for 187 days until July 2020 with a rather small trading volume of 3,748 tons, almost 98.25% less compared to the same period in 2019. Throughout 2020, total trading intervals without transactions amounted to 302 days on the Fujian Exchange.

The pilot markets suffered from great shocks during the pandemic, including markedly decreasing trading volumes and long-lasting transaction halts. Considering the negative coefficients of liquidity and continuity as in Table 8, neither shrinking volume nor discontinuity on pilot markets is a good sign for emission reductions, which could otherwise have been realized or augmented through better performances of ETS.

If regarded as a natural experiment, the pandemic, or indeed any crisis whatsoever, is intensely exposing the ever-existing defects of China's carbon ETS and its pilot markets. Low liquidity with trading volume usually concentrated right before compliance deadlines, few and non-diverse participants and their lack of incentives in emission trading, and regulated firms' tendency to reserve emission allowances for upcoming stricter policies rather than trading, as well as regional barriers among exchanges due to differential regulation and technical methods, have contribute to the weak market performances. When shocked by the pandemic, the markets would only perform even more vulnerably. Consequently, the ETS, as a key driver for emission reductions, might just end up with limited reduction effects. Unless handled properly with careful and effective mechanism design, the defects and immaturity of ETS markets would continuously undermine the emission reduction efforts in post-pandemic China.

## Conclusions

There have been shocks on the economic activities and carbon emissions all around the world since the outbreak of the pandemic. They could be only temporary, though, if there are no fundamental changes in the emission trajectory and its determinants by reason of the pandemic. To examine the CO_2_ emission trajectory for pre- and post-pandemic China, we use the near-real-time datasets of daily CO_2_ emissions by Carbon Monitor, and apply the Cox-Stuart trend test and mean equality test. A fairly consistent trajectory is observed over a 2-year time horizon before and after the pandemic outbreak, despite abrupt shocks in the first wave. Although for the time being we find no signs of significant changes in the emission trajectory, we believe it's necessary to further investigate the pandemic impacts on key forces underlying the trajectory and driving for emission reductions.

Amongst these drivers, particular importance has been attached to the market-based ETS. China's carbon ETS, however, has been working mostly through the eight pilot markets, which are immature and bound to undergo shocks during the pandemic. The effects on CO_2_ emissions by China's carbon ETS and its pilot market performances still need to be examined, especially when under pandemic shocks. To this end, we firstly estimate the reduction effects by ETS for pre-pandemic China, and then check on the pandemic impacts on pilot market performances with implications for emission reductions in post-pandemic China.

Using the methodology of PSM-DID and pre-pandemic region-level CO_2_ emission datasets by CEADs, we find statistically significant emission reduction effects by China's carbon ETS. Estimated with different sizes of matched samples using the algorithm of *k*-NNM within radius, CO_2_ emissions in pilot regions post the implementation of ETS are significantly reduced, with the average treatment effects ranging from 4–8.6%. For robustness checks, we apply two diagnostic tests for parallel trend across treatment and control groups, as well as a placebo test with 1,000 random permutations based on Monte Carlo simulation.

We also find statistically significant emission reduction effects by pilot market performances regarding the trading price, liquidity, and transaction continuity. The pilot markets are working in emission reductions through the fundamental signals of carbon prices, as well as the liquidity and continuous transactions of emission allowances. The pilot markets, however, have been experiencing great pandemic shocks. With markedly decreasing trading volumes and long-lasting transaction halts, the pilot markets have been performing more weakly and vulnerably under pandemic shocks compared to usual times. Consequently, the emission reduction effects by ETS during and after the pandemic could just end up undermined. Rather than being the root for such performance deterioration, however, the pandemic shocks are more like catalyzer, exposing the ever-existing defects on immature pilot markets intensely and aggravating the limited emission reductions by ETS.

If regarded as a natural experiment, just as previous crisis were, the pandemic might as well provide some lessons on the mechanism design of the carbon ETS for post-pandemic China. It's critical to ensure and maintain the market liquidity and transaction continuity, so as for the market-based ETS to fully realize its emission reduction effects. More efforts need to be devoted to increasing the trade varieties and introducing more and diverse participants into the markets. Encourage firms to get more involved in emission trading by the price discovery function of trading prices as they reflect the marginal abatement costs and provide incentives for emission trading, rather than just requiring them to meet control goals by compliances deadlines. Now that based on pilot operations, the nationwide market has entered into its first compliance cycle and started to play an increasingly core role in the carbon ETS, it's of particular value that we learn the lessons and improve the mechanism design of the national ETS for post-pandemic China. There are still a lot to be explored, especially regarding the desired properties of heterogeneous emission trading markets to the end of steadily reducing emissions even under shocks. We're to further our research, and expect to be in a better position to study with future access to more detailed datasets of CO_2_ emissions at all levels covering a longer time horizon.

## Data Availability Statement

Datasets of daily CO_2_ emission estimates can be accessed through Carbon Monitor (https://carbonmonitor.org.cn/). Refer to Liu et al. (4, 14) for technical details. CO_2_ emission inventories by regions of China using IPCC Sectoral Emission Accounting Approach can be accessed through CEADs (https://www.ceads.net/data/province/by_sectoral_accounting/). Refer to (37–40) for technical details. Datasets of emission transactions can be accessed through each pilot exchange, such as the Shanghai Environment and Energy Exchange (https://www.cneeex.com/), Beijing Green Exchange (https://www.cbeex.com.cn/), Guangdong China Emissions Exchange (http://www.cnemission.cn/), Tianjin Climate Exchange (https://www.chinatcx.com.cn/), China Hubei Emission Exchange (http://www.hbets.cn/), Chongqing Carbon Emissions Trading Center (https://tpf.cqggzy.com/), as well as the carbon emission trading platform (http://www.tanjiaoyi.org.cn/). Datasets for other variables used in this study can be accessed through China Statistical Yearbook, China Energy Statistical Yearbook, and China Statistical Yearbook on Environment, compiled by the NBS (National Bureau of Statistics, https://data.stats.gov.cn/).

## Author Contributions

KL: conceptualization, methodology, analysis, and writing. AX: conceptualization and reviewing. RY: methodology, analysis, and reviewing. WL: methodology, data analysis, and writing. All authors contributed to the article and approved the submitted version.

## Funding

This research was supported by National Social Science Foundation of China (Grant No. 21BTJ068) and Zhejiang Provincial Philosophy and Social Science Planning Project of China (Grant No. 22NDQN284YB).

## Conflict of Interest

The authors declare that the research was conducted in the absence of any commercial or financial relationships that could be construed as a potential conflict of interest.

## Publisher's Note

All claims expressed in this article are solely those of the authors and do not necessarily represent those of their affiliated organizations, or those of the publisher, the editors and the reviewers. Any product that may be evaluated in this article, or claim that may be made by its manufacturer, is not guaranteed or endorsed by the publisher.
